# Defining complex case management in the UK: a modified Delphi approach

**DOI:** 10.1136/bmjopen-2025-114962

**Published:** 2026-08-17

**Authors:** R Leonard, M Linden, The British Association of Brain Injury and Complex Case Management (BABICM), Andrew Patterson

**Affiliations:** 1 School of Social Sciences, Education and Social Work, Queen’s University Belfast, Belfast, UK; 2 Nursing and Midwifery, Queen’s University Belfast, Belfast, UK; 3 Phoenix House, Bury, UK

**Keywords:** Delphi Technique, Brain Injuries, Methods

## Abstract

**Abstract:**

**Objectives:**

To conduct a Delphi study to develop a consensus-based definition of complex case management within the UK context.

**Setting and participants:**

This study was conducted with members of the British Association of Brain Injury and Complex Case Management (BABICM).

**Design:**

A methodological approach informed by a modified Delphi design was employed, incorporating expert focus groups and two rounds of questionnaires distributed to members of the BABICM.

Initial focus groups (n=2) with eight expert case managers generated themes which were used to create a draft definition. Five definitions—four existing and one newly developed definition—were reviewed and refined through two rounds of online questionnaires. The top two definitions in round 1 were retained for round 2 (with no refinement required based on qualitative responses). The final definition was agreed-on when consensus was reached ≥80% agreement.

**Results:**

In round 1, 130 (10.8% of the 1200-member population) BABICM members ranked five definitions and provided qualitative feedback. Based on round 1 rankings, two definitions were retained for round 2. Definition 4, developed through expert focus groups, was ranked as the most preferred in round 1 (60%) and round 2 (85%), thus meeting the consensus threshold. The final definition emphasised collaborative, tailored support across systems, advocacy and person-centred care for clients and their families.

**Conclusions:**

This study provides a consensus-based definition of complex case management in the UK, reflecting the complexity and interdisciplinary nature of the role. The definition offers a foundation for standardising practice, informing training and guiding future research and policy development.

**STRENGTHS AND LIMITATIONS OF THIS STUDY**

- A key strength is the expert consultation in the development and implementation of the study.
- We used the Conducting and REporting DElphi Studies checklist to report this Delphi study.
- The use of a modified Delphi approach allowed for refinement of definitions based on expert participant feedback.
- The response rate of 10.8% in both rounds of the Delphi study.
- Reliance on self-reported data and potential non-response bias.

## Introduction

 Complex case management involves the coordination and integration of services and systems around individuals with complex needs and their families.^1–3^ Case managers work with individuals with a range of long-term or chronic injury or illness such as brain injury.^1 3 4^ These individuals often have complex needs due to the significant impairments to their functioning, which can hinder their ability to resume premorbid roles, responsibilities and occupations.^5^ To address the multifaceted and often long-term needs of the population they serve, specialist complex case management services have been developed to facilitate rehabilitation and improve outcomes.^1 2^

Case management remains a relatively new and evolving profession, with much of its development driven by practitioners themselves.^6^ There is currently a lack of a universally accepted definition of complex case management. This lack of an agreed-upon definition reflects the diversity of practice settings and the varied professional backgrounds of those working in the field.^4 7 8^ For example, case managers can work across acute and post-acute hospital care, rehabilitation services, long-term care facilities and community-based environments.^1 4^ Professional backgrounds also vary, with case managers coming from professions such as social work, nursing and occupational therapy.^1 4^ Although efforts have been made to define and standardise the concept,^1 4 9^ inconsistencies remain in how these definitions are applied both nationally and internationally. A recent review by Leonard *et al*^8^ highlighted the pressing need for a clear, consensus-based definition of complex case management that can be consistently implemented across research and practice.

This study therefore aimed to develop a consensus-based definition of complex case management within the UK context, using a Delphi methodology.

## Methods

### Study design

**Figure 1:**
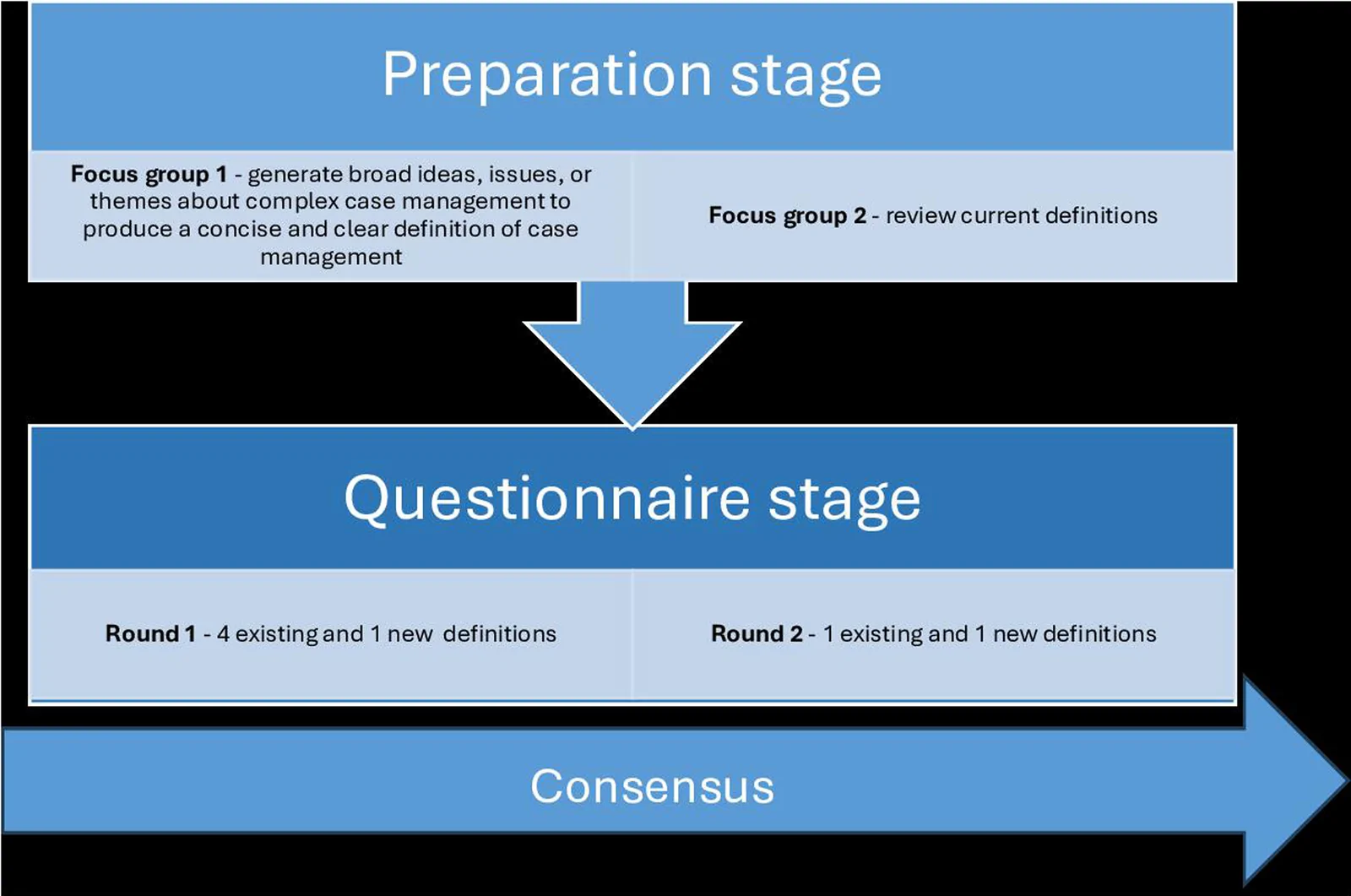
A modified Delphi process.

A modified Delphi method was deemed appropriate as this approach focuses on a complex topic and questions that require expertise and experience to answer.^10 11^ This method employs a panel of experts to gain consensus on the task through their feedback over several rounds of deliberations.^12^ This study was informed by a modified Delphi approach; however, it did not follow this technique rigidly. This study consisted of two stages; first, we conducted focus groups to review current definitions and generate ideas to develop a new definition of complex case management, with the aim of developing a questionnaire for stage 2. Second, based on the definitions decided on in stage 1, a questionnaire was distributed to rank definitions with the aim of reaching consensus on an agreed-upon definition of complex case management (see figure 1). A priori, consensus was defined as >80% agreement^11^ (see data analysis section for further detail). Across both stages, eligibility criteria for inclusion were British Association of Brain Injury and Complex Case Management (BABICM) membership and active case manager. The Conducting and REporting DElphi Studies (CREDES) checklist^13^ was used to demonstrate the quality of the study design (online supplemental data S1).

### Participants

Participants included all members of BABICM, with membership status as registered practitioner or advanced registered practitioner (ie, have a previous professional qualification as well as an accredited case manager qualification). BABICM is a membership organisation for professionals currently working in complex case management within the UK (see https://www.babicm.org/). Within the UK, case managers are largely privately funded or commissioned through litigation claims, however, this can occasionally be provided through the National Health Service.^14^ According to Avella,^11^ the number of participants on a Delphi panel is typically somewhere between 10 and 100, and there is no agreed standard. Eligibility criteria for inclusion across both stages were: BABICM membership and active case manager.

For stage 1, focus group participants comprised eight expert case managers selected by BABICM management, based on their ability to meaningfully contribute to the discussion. Expertise was determined by BABICM management, who are also registered case managers, which largely related to years of experience within case management. At this stage, we did not consider geographical spread or professional background of participants.

For stage 2, online questionnaires, using MS Forms, were distributed to all members of BABICM—approximately 1200 case managers. Round 2 of the questionnaire was also sent to the full cohort of 1200 BABICM case managers, which deviated from a typical Delphi approach.^11^

### Patient and public involvement

This study incorporated patient and public involvement throughout all stages of the study, in partnership with BABICM. Extensive consultation was conducted prior to data collection, between the research team and the BABICM to define and agree research goals and format of the study. The organisation supported recruitment, advised on inclusive communication and helped interpret interim findings by providing case managers’ perspectives that enriched the understanding of consensus patterns. BABICM members also reviewed the results and will support dissemination.

### Planned time frames

Consultations and planning began in January 2025 between the research team and BABICM. Expert members of the focus group were identified, and the first session took place in April 2025. Results from this focus group were analysed and a second session was arranged for May 2025. Following further refinement of definitions and agreement with the experts, the round 1 questionnaire was disseminated in July 2025. We initially allowed 2 weeks for responses; however, this was subsequently revised to 4 weeks based on poor response rates. Following analysis of the results from round 1 and refinement of the definitions (2 weeks), round 2 was sent at the end of August 2025. We sent one reminder to all potential participants for each questionnaire.

### Stage 1: focus groups

In preparing for the questionnaire rounds, we conducted two focus groups with a group of experts in case management. The aim of the focus groups was twofold: to (1) generate broad ideas, issues or themes about complex case management to produce a concise and clear definition of case management and (2) review current definitions. A ‘classical’ Delphi technique^11^ would have started with a blank sheet and asked the panellists to define a specific concept. A ‘modified’ Delphi technique^11^ can be used when a study has an advanced starting point. Our starting point was to bring existing definitions to the focus group in addition to the development of a new definition based on discussion. Existing definitions were based on a previous systematic review.^8^ However, this was an iterative process whereby if the expert participants in the focus group expressed that a current definition was not captured, the team would add that to discussion. Focus groups were conducted via Zoom and recorded. Notes were taken by one researcher (first author), while the other researcher (second author) facilitated discussion. Experts were identified by BABICM management. Members were not remunerated for their participation in the focus groups. To take part, participants had to be members of BABICM and have significant expertise in complex case management. Eight experts took part in the focus groups: five females and three males. All participants were case managers from England. The first focus group explored the role of complex case management, while the second explored current definitions. The first focus group also explored the overall aim of the Delphi study along with the specific aim of the session. The discussion began with a broad and open question: ‘What is your understanding of your role in complex case management’? Following the focus group, the research team analysed notes focusing on common themes. Eight themes were identified: specialist knowledge, a bridge between systems, the complexity of people’s lives, lifelong care, creativity and uniqueness of the role, required skills, education on the role of case management and challenges of case management. Based on these themes, the research team developed a draft definition of complex case management.

The second focus group sought to review the new definition, developed from the previous focus group and existing definitions. Group members were the same across both focus groups. The focus group reviewed five definitions (table 1), three existing definitions (from BABICM,^15^ Lukersmith *et al*^4^ and Case Management Society (CMS) Australia and New Zealand^9^) and two newly developed definitions from focus group one—a shortened version and a longer version.

**Table 1:** Reviewed definitions in focus group 2

Reviewed definition	Origin
Definition 1: ‘A collaborative process, which assesses, plans, implements, coordinates, monitors and evaluates the options and services required to meet an individual’s health and well-being, education and/or occupational needs, using communication and available resources to promote quality, cost-effective and safe outcomes’.	BABICM^15^
Definition 2: ‘Community-based case management is a multidimensional and collaborative process. It involves a set of interventions for assessment, planning, coordinating and review of the options and services required to meet the client’s health-related needs and support them to reach their goals related to participation in life roles’.	Lukersmith *et al*^4^
Definition 3: ‘Case management is a process, encompassing a culmination of consecutive collaborative phases that assist clients to access available and relevant resources necessary for the client to attain their identified goals. Key phases within the case management process include client identification (screening), assessment, stratifying risk, planning, implementation (care coordination), monitoring, transitioning and evaluation. Within the case management process the Case Manager navigates each phase of the case management process (as applicable) with careful consideration of the client’s individual, diverse and special needs, including aspirations, choices, expectations, motivations, preferences and values and available resources, services and supports’.	Case Management Society Australia and New Zealand^9^
Definition 4: ‘A Complex Brain injury Case Manager is an independent professional who collaboratively coordinates tailored, lifelong support for their clients. Bridging health, social care and legal systems, they use specialist knowledge to advocate for the client, navigate complex needs and deliver creative, person-centred solutions that enhance quality of life’.	Developed from expert group discussion (shortened version)
Definition 5: ‘A Complex Brain injury Case Manager is an independent professional who specialises in collaboratively coordinating and overseeing tailored, lifelong care for their clients. Working across health, social care, legal and community systems, they apply expert knowledge and an extensive professional network to advocate for the client’s best interests. Their role is to navigate complex challenges, deliver creative, person-centred solutions and support the client’s ongoing rehabilitation, independence and quality of life’.	Developed from expert group discussion (longer version)

BABICM, British Association of Brain Injury and Complex Case Management.

Definitions were presented to expert participants without reference to their origin and were discussed in depth in the second focus group. Based on discussions, the expert panel suggested several changes to the developed definitions (see table 2). The term complex brain injury case manager was amended to ‘complex case management’ to acknowledge that the definition encompasses the overall function and process, not just the professional title. In addition, the expert panel acknowledged that at times their clients have multiple conditions that may or may not include brain injury. Therefore, the term ‘brain injury’ was removed. The expert panel suggested the term ‘lifelong’ be removed, given that case management can also be short term. The phrase ‘independent professional’ was removed as it was felt that this did not accurately reflect the role of a case manager who often worked interprofessionally. Lastly, the term ‘families’ was added, to reflect that case management involves support to both the client and family. The group discussed both the short and long versions of the developed definition and agreed (100% consensus) that the longer version was preferable (definition 4). Participants were asked whether any relevant definitions had been overlooked or excluded from discussion. The group discussed an older version of a BABICM^14^ definition (definition 5) which they felt was useful. It was therefore agreed that five definitions would be retained for round 1 questionnaires (table 2).

**Table 2:** Definitions retained for round 1 and changes made

Definitions retained for round 1	Origin and summary of changes made
Definition 1: ‘A collaborative process, which assesses, plans, implements, coordinates, monitors and evaluates the options and services required to meet an individual’s health and well-being, education and/or occupational needs, using communication and available resources to promote quality, cost-effective and safe outcomes’.	BABICM^15^ No changes made
Definition 2: ‘Community-based case management is a multidimensional and collaborative process. It involves a set of interventions for assessment, planning, coordinating and review of the options and services required to meet the client’s health-related needs, and support them to reach their goals related to participation in life roles’.	Lukersmith *et al*,^4^ No changes made
Definition 3: ‘Case management is a process, encompassing a culmination of consecutive collaborative phases that assist clients to access available and relevant resources necessary for the client to attain their identified goals. Key phases within the case management process include: client identification (screening), assessment, stratifying risk, planning, implementation (care coordination), monitoring, transitioning and evaluation. Within the case management process the Case Manager navigates each phase of the case management process (as applicable) with careful consideration of the client’s individual, diverse and special needs, including aspirations, choices, expectations, motivations, preferences and values and available resources, services and supports’.	CMS Australia and New Zealand^9^ No changes made
Definition 4: 'Complex case management specialises in collaboratively coordinating and overseeing tailored support for clients and their families. Working across health, social care, legal and community systems, they apply expert knowledge and an extensive professional network to advocate for the client’s best interests. Their role is to navigate complex challenges, deliver creative, person-centred solutions, to support the client’s ongoing rehabilitation, independence and quality of life’.	Developed from expert group discussion ‘Complex brain injury case manager’ changed to ‘complex case management’. ‘Lifelong care’ changed to ‘support’. ‘Independent professional’ removed. ‘And their families’ added.
Definition 5: ‘An active process devoted to the coordination, rehabilitation, care and support of people with complex, clinical needs and their families. It aims to facilitate their independence and improve their quality of life while acknowledging safety issues’.	BABICM^14^ No changes made

BABICM, British Association of Brain Injury and Complex Case Management; CMS, case management society.

### Data collection: stage 2 questionnaire

There were two rounds of online questionnaires (online supplemental data S2). The first questionnaire included five definitions of complex case management (four existing and one new—table 2). Participants were asked to rank the definitions from most to least preferred (with five the most preferred and one the least). The first questionnaire included open text comment boxes to allow participants to provide feedback on why they ranked definitions in a particular order (ie, parts they liked or disliked in each definition). The responses to these open-ended questions were reviewed; however, no revisions were required to any definition. Demographic information, including age, gender, region of the UK, professional background and years of experience (as a case manager) were also collected. Following analysis of round 1 results, definitions were reviewed and included in the round 2 questionnaire. The second questionnaire followed the same format; however, only included two of the highest ranked definitions from round 1. In addition, feedback to participants from round 1 was not given to participants before round 2. Potential participants for both rounds included all members of BABICM—approximately 1200 case managers. Rather than restricting round 2 to prior participants, the questionnaire was sent to the full cohort of 1200 BABICM case managers. These approaches deviated from a typically Delphi approach.^11^

### Data analysis: stage 2 questionnaire

Descriptive statistics were used to summarise participant demographics. For each round, participants rated the five proposed definitions of Complex Case Management. Percentages were used to calculate consensus (percentage of participants who ranked each definition as their most preferred vs least preferred; ie, 1–5). The top two most highly ranked definitions were retained for round 2, while the others were removed. Reaching consensus in a Delphi study is the primary aim; however, there is some inconsistency within the literature about how consensus is determined, with levels ranging from 51% to 100%.^12^ A systematic review of Delphi studies found that consensus was typically agreed-on between 70% and 80%.^16 17^ The current study determined consensus to be met whereby >80% of experts reached agreement on a particular definition (by the end of round 2). These were then re-rated in subsequent rounds until stability in responses was observed.

## Results

### Demographics and response rate

Demographics and response rate reported related to the questionnaire completed in stage 2 of this study. Details on the participants with the focus groups are reported previously. The round 1 questionnaire received 130 responses from the total population of BABICM members (n=1200, 10.8% response rate). Round 2 also had 130 responses (10.8% response rate). Both sets of questionnaires were distributed to the complete population of 1200 case managers at BABICM. Therefore, there were some differences between the samples for both rounds of questionnaires (ie, the 130 were not the exact same participants in both rounds). The demographics for each round are detailed below.

170 females and 12 males responded to round 1, with a mean age of 50 years (SD=9.44). The majority of respondents were from England (n=118, 91%), with few from Scotland (n=5, 4%) and Wales (n=7, 5%). Most respondents had a background in occupational therapy (n=50, 38%), with others coming from nursing (n=35, 27%), social work (n=13, 10%) and physiotherapy (n=9, 7%). Respondents had an average of 11.5 years of experience as a case manager (SD=8.78) (see table 3).

**Table 3:** Demographics of respondents to round 1 questionnaire

Demographic	
Total	N=130
Age	Mean=50 years (SD=9.44) Min=28 years, Max=71
Gender	Male=12 (9%) Female=117 (90%) Prefer not to say=1 (1%)
Region	England=118 (91%) Scotland=5 (4%) Wales=7 (5%)
Professional background	Occupational therapist=50 (38%) Nurse=35 (27%) Social worker=13 (10%) Physiotherapist=9 (7%) Other=23 (18%)
Years as a case manager	Mean=11.5 years (SD=8.78) Min=1, Max=41

In round 2, 130 case managers completed the questionnaire. This included 114 females and 13 males, with a mean age of 51 years (SD=7.96). The majority of respondents were from England (n=115, 88%), with few from Scotland (n=6, 5%), Wales (n=5, 4%) and Northern Ireland (n=4, 3%). Most respondents had a background in occupational therapy (n=51, 39%), with others coming from nursing (n=35, 27%), social work (n=15, 12%) and physiotherapy (n=10, 8%). Respondents had an average of 11.9 years of experience as a case manager (SD=8.08) (see table 4).

**Table 4:** Demographics of respondents to round 2 questionnaire

Demographic	
Total	N=130
Age	Mean=51 years (SD=7.96) Min=30 years, Max=71
Gender	Male=13 (10%) Female=114 (88%) Prefer not to say=3 (2%)
Region	England=115 (88%) Scotland=6 (5%) Wales=5 (4%) Northern Ireland=4 (3%)
Professional background	Occupational therapist=51 (39%) Nurse=35 (27%) Social worker=15 (12%) Physiotherapist=10 (8%) Other=19 (14%)
Years as a case manager	Mean=11.9 years (SD=8.08) Min=1, Max=35

### Results from round 1

There were five definitions included in the round 1 questionnaire:

- Definition 1: BABICM.^15^
- Definition 2: Lukersmith *et al.*^4^
- Definition 3: CMS Australia and New Zealand.^9^
- Definition 4: Developed through focus group consultation.
- Definition 5: BABICM.^14^

**Figure 2:**
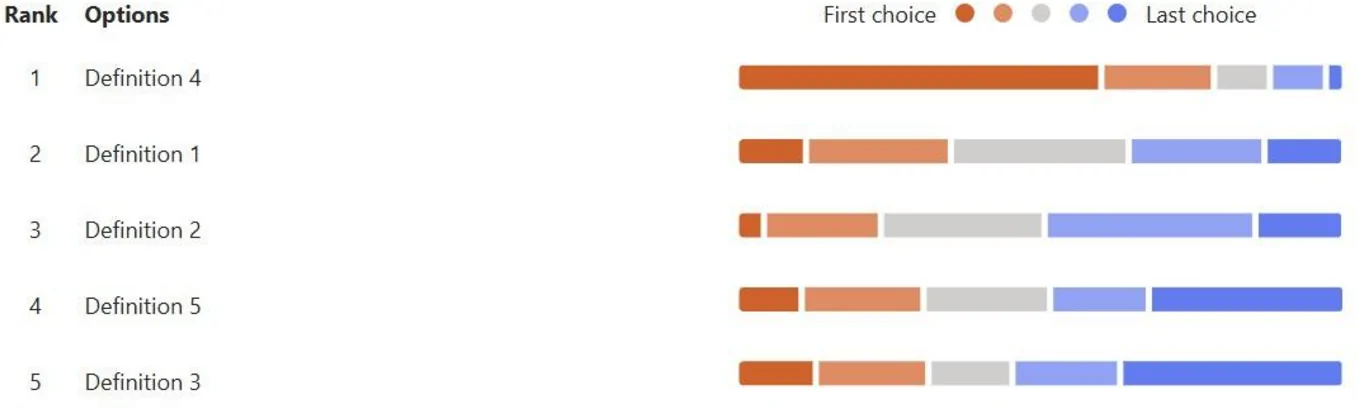
Findings from round 1 questionnaire ranking here.

Results from expert ranking (see figure 2) suggested that definition 4 (developed through expert focus group) was the most preferred option (60% ranked as first choice). With definition 3 (CMS Australia and New Zealand) being the least preferred (37% ranked as last choice). Definition 1^15^ was ranked by 24% of participants as their second choice. Following review of the qualitative comments, no further changes were made to any definitions. Based on the results, the team agreed to retain definitions 4 and 1 for round 2.

### Results from round 2

In round 2, expert participants were asked to rank the following two definitions:

- Definition 1: BABICM.^15^
- Definition 4: Developed through focus group.

**Figure 3:**
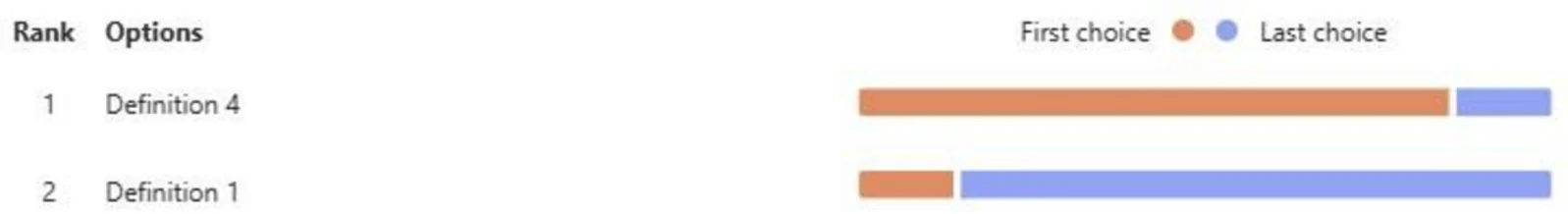
Findings from round 2 questionnaire ranking.

Definition 4 was the most preferred, with 85% ranking this as their first choice (see figure 3). Definition 1 was therefore the least preferred, with 15% ranking this as their first choice. Based on the a priori criteria for consensus (>80%), it was concluded that consensus had been met and experts agreed that definition 4 was the most preferable description of complex case management.

## Discussion

This Delphi study sought to achieve consensus on a definition of complex case management in the UK, addressing an identified gap in this area. This iterative process involved expert focus groups and two rounds of questionnaires. Results showed that 85% of participants selected the new expert derived definition as their preferred choice, thus exceeding the consensus threshold of >80%.

In practice settings, an agreed-on definition promotes consistency in implementation, communication and evaluation.^8^ It ensures that practitioners, policymakers and service users share a common understanding of key concepts, reducing misinterpretation and variation in delivery. This clarity supports fidelity to interventions, enables more accurate monitoring of outcomes and strengthens accountability mechanisms. Standardised definitions also streamline training, onboarding and cross-sector coordination, particularly in multiagency environments, by providing a stable reference point for guidance and decision-making.^18^

An agreed-on definition strengthens research by enhancing conceptual clarity, methodological consistency and cross-study comparability. It enables researchers to operationalise constructs with precision, improving the validity and reliability of measurements.^3 4^ Shared definitions also facilitate synthesis across studies, particularly in systematic reviews and meta-analyses, by reducing semantic ambiguity and allowing for meaningful pooling of findings. In interdisciplinary contexts, consensus definitions bridge disciplinary language barriers, fostering collaboration and cumulative knowledge-building.^19^ Moreover, they support transparent reporting and reproducibility, which are essential for advancing evidence-based practice and policy.

The preferred definition was developed through expert focus groups with case managers. It was derived from their discussion of their role in complex case management and reflections on current definitions. The consensus-based definition reflects the multifaceted nature of complex case management, emphasising coordination across health, social care, legal and community systems, and the importance of advocacy, creativity and person-centred approaches: *‘*Complex case management specialises in collaboratively coordinating and overseeing tailored support for clients and their families. Working across health, social care, legal and community systems, they apply expert knowledge and an extensive professional network to advocate for the client’s best interests. Their role is to navigate complex challenges, deliver creative, person-centred solutions, to support the client’s ongoing rehabilitation, independence and quality of life’. Notably, the inclusion of families in the definition acknowledges the broader impact of conditions and the holistic support required. The removal of terms such as ‘lifelong’ and ‘independent professional’ demonstrates the expert panel’s nuanced understanding of the variability and context-specific nature of case manager’s roles.

These findings align with previous literature highlighting the diversity and evolving nature of case management,^3 8^ and reinforce the need for a standardised, contextually relevant definition to guide practice, training and research. The consensus-based definition offers a foundation for future policy development and professional standards within the UK and potentially beyond. Importantly, this study has solely considered the perspectives of case managers from one UK organisation. The perspectives of other stakeholders are equally important in the development of a consensus-based definition. Future research should continue the development of the agreed definition within this current study, and consider the perspectives of stakeholders such as, patients, families and other case management organisations.

### Strengths and limitations

A key strength of this study is its rigorous methodology, including expert consultation, use of the CREDES checklist and a modified Delphi approach that allowed for refinement of definitions based on expert participant feedback. The inclusion of a broad range of case managers from different professions across the UK enhances the generalisability of the findings.

However, the study is not without limitations. While our sample size of 130 in both rounds of questionnaires is consistent with similar Delphi study sample sizes,^11^ reflecting a 10.8% response rate. This may limit the representativeness of the findings. In addition, due to the questionnaire being distributed to all 1200 members of BABICM, the 130 participants in both rounds of the questionnaire were not the exact same sample. This differs from a classical Delphi approach, which typically involves the same panel reconsidering their responses in light of group trends. Our approach is informed by a modified Delphi approach, however, we did not apply this approach rigidly. Namely, the difference in round 1 and round 2 samples, and anonymised feedback to participants from round 1 before round 2 was not provided. While this means the results of the questionnaire are not from a single iterative panel, the high level of agreement across rounds still indicates a strong collective preference for definition 4.

Additionally, while this study is a UK wide study, we acknowledge that the majority of our sample was from England. Therefore, findings may not reflect variations in case management perspectives across various regions in the UK. The reliance on self-reported data and potential non-response bias^20^ should be considered when interpreting results. Lastly, the authors note the potential for organisational influence, given the role BABICM had in funding this study, the selection of focus group participants and as an author on this paper.

## Conclusions

This study has successfully developed a consensus-based definition of complex case management in the UK, through a rigorous Delphi process with expert case managers. By establishing a shared understanding, this definition provides a foundation for greater consistency in practice, training and research. A shared understanding of complex case management not only enhances clarity across professional boundaries but also strengthens the identity and credibility of case management as a distinct discipline. It enables more coherent communication among practitioners, supports the development of targeted education and training programmes and facilitates more robust evaluation of service models. Ultimately, such alignment can contribute to improved interdisciplinary collaboration and better outcomes for those receiving care. Future work should focus on embedding this definition into professional standards and evaluating its impact on service delivery and outcomes for individuals and their families.

## Supplementary material

10.1136/bmjopen-2025-114962 online supplemental file 1

10.1136/bmjopen-2025-114962 online supplemental file 2

## Data Availability

All data relevant to the study are included in the article or uploaded as supplementary information.
