# Supplementary material for: Defining complex case management in the UK: a modified Delphi approach
Source: BMJ Open. 2026 Aug 17;16(8):e114962. doi: 10.1136/bmjopen-2025-114962 (PMC13504933; doi:10.1136/bmjopen-2025-114962)
Supplement: Supplementary file 2 [file bmjopen-2025-114962.reviewer_comments.pdf]

PEER REVIEW HISTORY

BMJ Open publishes all reviews undertaken for accepted manuscripts. Reviewers are asked to complete a checklist review form and are provided with free text boxes to elaborate on their assessment. These free text comments are reproduced below.

ARTICLE DETAILS

Title (Provisional)

Defining Complex Case Management in the UK: A Modified Delphi Approach

Authors

Leonard, R; Linden, M; The British Association of Brain Injury, Complex Case Management (BABICM)

VERSION 1 - REVIEW

**Reviewer**	**1**
**Name**	**Fong, Nicholas**
**Affiliation**	**UCSF, Anesthesia and Perioperative Medicine**
**Date**	**06-Feb-2026**
**COI**	**None**

Dear authors,

Your paper reports a modified Delphi study to establish a consensus definition of Complex Case Management for the UK. Focus groups with eight expert case managers generated draft definitions, which were then put to BABICM's membership (~1,200 case managers) alongside existing definitions across two questionnaire rounds (n=130 each). The expert-derived definition was preferred by 85% of round two respondents, meeting the pre-specified consensus threshold of >80%.

I want to thank the authors for the opportunity to review your manuscript. The authors make a reasonable case, supported by their cited sources, that definitional ambiguity in case management creates practical problems for training, research, and cross-sector communication. I am raising the concerns below because I think they are addressable, and working through them would strengthen the manuscript. I would be happy to review a revised version.

1. Both questionnaires went out to the full BABICM membership, and the authors note that "there were two different samples for both rounds of questionnaires." I want to flag that it is possible I am misreading the design here, and if so I would welcome clarification. But as I understand it, round two participants were not necessarily the same individuals as round

one participants, and were not shown the aggregated group feedback from round one before responding. If that is correct, I think it is worth addressing carefully, because the iterative logic of the Delphi method depends on the same panellists seeing group-level results and reconsidering their positions. Without that loop, what the study shows is that Definition 4 was the most popular choice in two independent cross-sectional samples, which is still a meaningful finding, but sits differently than Delphi-derived consensus as it is conventionally understood. I would find it helpful if the authors either pointed to methodological precedent for this approach or adjusted the framing to more precisely describe what was achieved. If I have misunderstood the sampling strategy, a small clarification in the methods section would likely prevent other readers from making the same mistake.

2. The methods section says qualitative feedback from round one was used to refine the two retained definitions for round two, but as far as I can tell, the paper does not show what those refinements were. We do not see the qualitative data (even in summary), and we do not see the pre- and post-revision wording side by side. I believe this matters because the iterative refinement is arguably the most important part of the Delphi process. If the revisions were minor, then round two is essentially a confirmation vote, which is fine, but it would be worth stating that explicitly. If they were substantial, then the 85% consensus figure applies to a somewhat different definition than the one that scored 60% in round one. I think including this information, perhaps in a supplementary table, would be straightforward and would add a lot of transparency.

3. I believe there is an inconsistency between the appendix and the main text. Definition 4 in the supplementary questionnaire begins with "Brain injury and Complex case management," but Table 2 states that "brain injury" was removed after the focus groups. Perhaps I am misunderstanding, but this seems inconsistent.

4. I also believe the abstract may miscount the definitions. It refers to "three existing and two versions of a developed definition," but unless I am misreading the sequence, round one included four existing definitions (BABICM 2018, Lukersmith et al. 2016, CMS Australia/New Zealand 2017, and BABICM 2013) plus one newly developed definition, since the BABICM 2013 version was added after the second focus group. If I have this right, it is a small fix.

5. The authors cite Avella (2016) to note that 10.8% is "consistent with similar Delphi study sample sizes," but I believe this may conflate two separate issues. A panel of 130 is a perfectly adequate size for a Delphi study. My concern is not how many people responded, but that nearly 90% of the eligible membership did not. That raises a different question: whether the 130 who did respond are representative of the broader BABICM membership, or whether they differ in ways that might matter (e.g., professional background, years of experience, level of engagement with the organisation). A brief acknowledgment of this distinction in the limitations section could help.

6. I believe BABICM's multiple roles should be more explicitly acknowledged in the limitations section. BABICM funded the study, selected the focus group experts, distributed the questionnaires, and appears as a group author. The funding disclosure is clearly stated, which I appreciate. A brief note in the limitations section recognizing the potential for organizational influence, even if unintentional, would round this out.

Thank you again for the opportunity to review your work.

Reviewer **2**

Name **Godbolt, Alison**

Affiliation **Karolinska Institute & Danderyd Hospital, Dept of Clinical Sciences and University Dept of Rehabilitation Medicine**

Date **15-Feb-2026**

COI **None**

This article addresses definitions of Complex Case Management for Brain Injury in the UK. This is a topic of importance to survivors of brain injury and their families and consensus on definitions would aid developments in the field.

There are, however, some limitations and areas that are insufficiently described, so revision would be needed before publication could be considered.

Title:

The title makes it clear that the purpose was defining complex case management for Brain Injury. Indeed, the work has been initiated by a Brain Injury Association with contributions from its members, and as such is clearly performed from a brain injury perspective. As such it is a little strange that the final consensus definition does not mention brain injury. Establishment of a broader definition of complex case management that is not diagnosis specific would need to include experiences from case managers focusing on other diagnoses.

It is also debatable as to whether a truly UK perspective is given: the focus group members were all from England and fewer than 10% of questionnaire respondents were from each of Scotland, Wales and Northern Ireland.

Setting:

More information is needed about the British Association of Brain Injury and Complex Case Management (BABICM). Readers, including international readers, need to be able to understand the setting of the study, especially as the study is funded by the BABICM and BABICM members are respondents to the questionnaires. For example, who can become a member? How is the organisation funded? What is its purpose and

professional / organisational anchoring? Information and perhaps a link to a website would help. Also, some basic information on brain injury case management in the UK would be relevant. For example: by whom are most case managers employed? Do some patients have publicly funded case managers, or are they usually privately engaged by the patient/family? Do criteria exist regarding which patients are eligible for a case manager?

“Strengths and limitations”

Suggest making it a little clearer which points are strengths and which limitations. Revision is likely to be needed once the article has been revised.

Methods:

Generally, information on participants is spread out between different sections, which makes it rather hard to find key points.

There is some lack of clarity regarding the study design and application of Delphi terminology: The BABICM management selected the expert participants for the focus groups, which (together with the absence of explicit criteria for panel selection) could be a source of bias and should be commented on. It also introduces some lack of clarity as to whether the BABICM management or the other authors are the “convenors” in the Delphi sense. The focus groups are an interesting and relevant preparatory step, but describing the focus group members after the questionnaire respondents under “participants” introduces a lack of clarity about the Delphi process. The text should be adjusted so that the reader cannot mistake the focus groups for the panel.

Some more information on focus group selection would aid transparency and increase understanding of the context. Were there other criteria considered in addition to the two that are stated (membership of BABICM and significant expertise in complex case management)? How was “significant expertise” operationalized? Other selection criteria for the focus groups could have been considered (for example range of locations, rural/cosmopolitan representation, range of professions and/or employers), and it would be relevant to understand if these aspects were considered or not.

Information about any conflicts of interest for members of the focus group should be included. Were focus group members renumerated for their participation by the BABICM?

In the CREDES checklist (supplemental information), the authors refer to pages 8-9 for a flow chart for the Delphi process, but I can’t find such a flow chart and wonder if they are referring to the text. If so, it needs to be clearer.

Under “participants” (page 6) and “results” (abstract, page 4) it is implied that the respondents to the questionnaires (i.e. not the focus groups) are the Delphi expert panel. This is problematic as the pool of possible respondents (1200) is too large to

reasonably come within definitions of Delphi panels. The description in the abstract does not make it clear that the 130 respondents are in fact only around 10% of potential respondents, and the requirements for participating by responding to the questionnaire are not really compatible with being described as an expert panel: it seems unlikely that all 1200 members of the organisation would be experts). The "panel" seems as such to be rather ad hoc, defined after the event as those responding to at least one round of questionnaires. The only requirements for responders were BABICM membership and "active case management". What is required for BABICM membership? How did the authors assess whether respondents met the criterion "active-case management"?

As the respondents to the second questionnaire were not the same as the respondents to the first round, the iterative Delphi process has not really been applied in its intended sense, which needs to be more clearly addressed as a limitation. Also, the question on "years of experience" is a little unclear and open to variable interpretation. Is it years of experience within the respondent's profession that is meant, or specifically years of experience as a case manager?

I can't see that the abbreviation QUB has been defined (page 6). This should be rectified.

A brief description of data analysis after download from MS forms should be included.

The methodology behind the selection (or exclusion) of definitions presented to the focus groups needs to be described. For example, was selection based on a previous review, or on database searches, or something else?

One limitation of the questionnaire is that whilst participants ranked the definitions in order of preference, there does not seem to have been a question as to whether respondents actually endorsed the top ranked definition, only that it was the most preferred of the definitions presented. One could argue that this gives sufficient proxy for reaching consensus, but it's not quite the same as >=80% of respondents giving a clear endorsement.

Results:

18% of respondents had a professional background of "other". This is missing from the text. What were these "other" professional backgrounds?

Discussion and conclusions:

A discussion of the need for other perspectives on the definition of complex case management is not included. This article gives the perspective of professionals working as complex case managers within brain injury, but other stakeholders (patients, families, employers, agencies) have not had the opportunity to give their views or

present possible alternative existing definitions. Neither have complex case managers outside membership of this brain injury organisation had the opportunity to participate. A broader dialogue would seem to be needed as a next step, and such points would usefully be included as limitations or next steps.

It would also be relevant to see more discussion of the specific needs of brain injury survivors in the context of case management, and/or why some patient groups have a need for a case manager, whilst others do not. Cognitive, behavioural and other impairments after brain injury can make it impossible for the individual themselves to drive the necessary contacts and processes with multiple agencies, and information on such aspects would aid understanding.

VERSION 1 - AUTHOR RESPONSE

Reviewer comments	Response
**Reviewer 1**	
Your paper reports a modified Delphi study to establish a consensus definition of Complex Case Management for the UK. Focus groups with eight expert case managers generated draft definitions, which were then put to BABICM's membership (~1,200 case managers) alongside existing definitions across two questionnaire rounds (n=130 each). The expert-derived definition was preferred by 85% of round two respondents, meeting the pre-specified consensus threshold of >80%.	
I want to thank the authors for the opportunity to review your manuscript. The authors make a reasonable case, supported by their cited sources, that definitional ambiguity in case management creates practical problems for training, research, and cross-sector communication. I am raising the concerns below because I think they are addressable, and working through them would strengthen the manuscript. I would be happy to review a revised version.	Thank you for your comments and for taking the time to review our paper.
1. Both questionnaires went out to the full BABICM membership, and the authors note that "there were two different samples for both rounds of questionnaires." I want to flag that it is possible I am misreading the design here, and if so I would welcome clarification. But as I understand it, round two participants were not necessarily the same individuals as round one participants, and were not shown the aggregated group feedback from round	Thank you for your comment – we have now added a section within the limitations to highlight these differences in the samples. We have also drawn attention to the point that the 130 participants are not exactly the same however they are all from the same target sample of case managers. We hope that these revisions will aid in transparency.

one before responding. If that is correct, I think it is worth addressing carefully, because the iterative logic of the Delphi method depends on the same panellists seeing group-level results and reconsidering their positions. Without that loop, what the study shows is that Definition 4 was the most popular choice in two independent cross-sectional samples, which is still a meaningful finding, but sits differently than Delphi-derived consensus as it is conventionally understood. I would find it helpful if the authors either pointed to methodological precedent for this approach or adjusted the framing to more precisely describe what was achieved. If I have misunderstood the sampling strategy, a small clarification in the methods section would likely prevent other readers from making the same mistake.	
2. The methods section says qualitative feedback from round one was used to refine the two retained definitions for round two, but as far as I can tell, the paper does not show what those refinements were. We do not see the qualitative data (even in summary), and we do not see the pre- and post-revision wording side by side. I believe this matters because the iterative refinement is arguably the most important part of the Delphi process. If the revisions were minor, then round two is essentially a confirmation vote, which is fine, but it would be worth stating that explicitly.<br>If they were substantial, then the 85% consensus figure applies to a somewhat different definition than the one that scored 60% in round one. I think including this information, perhaps in a supplementary table, would be straightforward and would add a lot of transparency.	Thank you for your observation. We have revised the methods and results to highlight that qualitative comments were collected to make changes if required. However following review of these comments in round 1 no additional revisions were made to any definition.
3. I believe there is an inconsistency between the appendix and the main text. Definition 4 in the supplementary questionnaire begins with "Brain injury and Complex case management," but Table 2 states that "brain injury" was removed after the focus groups. Perhaps I am misunderstanding, but this seems inconsistent.	Thank you – we have now correct this in the supplementary file.
4. I also believe the abstract may miscount the definitions. It refers to "three existing and	We have now corrected this in the abstract.

two versions of a developed definition," but unless I am misreading the sequence, round one included four existing definitions (BABICM 2018, Lukersmith et al. 2016, CMS Australia/New Zealand 2017, and BABICM 2013) plus one newly developed definition, since the BABICM 2013 version was added after the second focus group. If I have this right, it is a small fix.	
5. The authors cite Avella (2016) to note that 10.8% is "consistent with similar Delphi study sample sizes," but I believe this may conflate two separate issues. A panel of 130 is a perfectly adequate size for a Delphi study. My concern is not how many people responded, but that nearly 90% of the eligible membership did not. That raises a different question: whether the 130 who did respond are representative of the broader BABICM membership, or whether they differ in ways that might matter (e.g., professional background, years of experience, level of engagement with the organisation). A brief acknowledgment of this distinction in the limitations section could help.	Thank you for your comment – we have now added the following to our limitations: "While our sample size of 130 in both rounds of questionnaires is consistent with similar Delphi study sample sizes (Avella, 2016), this was reflective of a 10.8% response rate. This may limit the representativeness of the findings".
6. I believe BABICM's multiple roles should be more explicitly acknowledged in the limitations section. BABICM funded the study, selected the focus group experts, distributed the questionnaires, and appears as a group author. The funding disclosure is clearly stated, which I appreciate. A brief note in the limitations section recognizing the potential for organizational influence, even if unintentional, would round this out.	We have now added the following to the limitations section – "Lastly, the authors note the potential for organisational influence, given the role BABICM had in both funding this study and in its design and data collection."
**Reviewer 2**	
This article addresses definitions of Complex Case Management for Brain Injury in the UK. This is a topic of importance to survivors of brain injury and their families and consensus on definitions would aid developments in the field.<br>There are, however, some limitations and areas that are insufficiently described, so revision would be needed before publication could be considered.	Thank you for your comments and for taking the time to review our paper.
Title: The title makes it clear that the purpose was defining complex case management for Brain Injury. Indeed, the work has been initiated by a Brain Injury Association with contributions from its members, and as such	Thank you for your comment. We have now removed the term "brain injury" from the title of the paper. The reason that brain injury is removed from definitions is based on expert feedback from case managers. The expert

is clearly performed from a brain injury perspective. As such it is a little strange that the final consensus definition does not mention brain injury. Establishment of a broader definition of complex case management that is not diagnosis specific would need to include experiences from case managers focusing on other diagnoses. It is also debatable as to whether a truly UK perspective is given: the focus group members were all from England and fewer than 10% of questionnaire respondents were from each of Scotland, Wales and Northern Ireland.	panel acknowledged that at times their clients have multiple conditions that may or may not include brain injury. Therefore, the term "brain injury" was removed. Given the study was aimed at reaching expert consensus this was deemed an appropriate action based on expert consensus. However, consensus does not mean that all case managers will agree with this removal.<br><br>Thank you for your comment about representativeness – we have added the following to the limitations section "Additionally, while this study is a UK wide study, we acknowledge that the majority of our sample was from England. Therefore, findings may not reflect variations on case management perspective across various regions in the UK."<br>It is also important to note that NI has a low number of case managers compared to other regions in the UK -therefore this may explain the low numbers.
Setting: More information is needed about the British Association of Brain Injury and Complex Case Management (BABICM). Readers, including international readers, need to be able to understand the setting of the study, especially as the study is funded by the BABICM and BABICM members are respondents to the questionnaires. For example, who can become a member? How is the organisation funded? What is its purpose and professional / organisational anchoring? Information and perhaps a link to a website would help. Also, some basic information on brain injury case management in the UK would be relevant. For example: by whom are most case managers employed? Do some patients have publicly funded case managers, or are they usually privately engaged by the patient/family? Do criteria exist regarding which patients are eligible for a case manager?	We have now added additional setting information to the participants section to give some context for readers around BABICM.
"Strengths and limitations" Suggest making it a little clearer which points are strengths and which limitations. Revision is likely to be needed once the article has been revised.	We have broken the strengths and limitations into two paragraphs.
Methods: Generally, information on participants is spread out between different	The paper has a section titled "participants" which details who participants were and how many etc. We are open to suggestions on

sections, which makes it rather hard to find key points.	what other details to add to this section to make this clearer.
There is some lack of clarity regarding the study design and application of Delphi terminology: The BABICM management selected the expert participants for the focus groups, which (together with the absence of explicit criteria for panel selection) could be a source of bias and should be commented on. It also introduces some lack of clarity as to whether the BABICM management or the other authors are the "convenors" in the Delphi sense. The focus groups are an interesting and relevant preparatory step, but describing the focus group members after the questionnaire respondents under "participants" introduces a lack of clarity about the Delphi process. The text should be adjusted so that the reader cannot mistake the focus groups for the panel.	We have now revised the participant section and the study design section to add some clarity to the two stages and two participant groups in this study.
Some more information on focus group selection would aid transparency and increase understanding of the context. Were there other criteria considered in addition to the two that are stated (membership of BABICM and significant expertise in complex case management)? How was "significant expertise" operationalized? Other selection criteria for the focus groups could have been considered (for example range of locations, rural/cosmopolitan representation, range of professions and/or employers), and it would be relevant to understand if these aspects were considered or not.	Thank you for your comments – we have now added additional information on the context and selection of focus group participants to help aid transparency.
Information about any conflicts of interest for members of the focus group should be included. Were focus group members renumerated for their participation by the BABICM?	Thank you – we have added additional detail in the limitations on conflicts of interests based on reviewer 1 comment.<br><br>We have also added the following "Members were not remunerated for their participants in the focus groups."
In the CREDES checklist (supplemental information), the authors refer to pages 8-9 for a flow chart for the Delphi process, but I can't find such a flow chart and wonder if they are referring to the text. If so, it needs to be clearer.	We have now added Figure 1 which details the modified Delphi process. We hope this will add further clarity to the study design.
Under "participants" (page 6) and "results" (abstract, page 4) it is implied that the respondents to the questionnaires (i.e. not the focus groups) are the Delphi expert panel.	We have made extensive changes in both the methods and results to highlights the differences in the two groups of participants.

This is problematic as the pool of possible respondents (1200) is too large to reasonably come within definitions of Delphi panels. The description in the abstract does not make it clear that the 130 respondents are in fact only around 10% of potential respondents, and the requirements for participating by responding to the questionnaire are not really compatible with being described as an expert panel: it seems unlikely that all 1200 members of the organisation would be experts). The "panel" seems as such to be rather ad hoc, defined after the event as those responding to at least one round of questionnaires. The only requirements for responders were BABICM membership and "active case management". What is required for BABICM membership? How did the authors assess whether respondents met the criterion "active-case management"?	We have added into the abstract that 130 participants were out of the potential 1200 BABICM members.
As the respondents to the second questionnaire were not the same as the respondents to the first round, the iterative Delphi process has not really been applied in its intended sense, which needs to be more clearly addressed as a limitation. Also, the question on "years of experience" is a little unclear and open to variable interpretation. Is it years of experience within the respondent's profession that is meant, or specifically years of experience as a case manager?	Thank you for your comment. We have added detail in the limitations regarding the samples for both rounds in the questionnaire. We have also discussed this point above under reviewer one's comment.<br><br>Within the tables and results we have specified that years of experiences refers to years as a case manager.
I can't see that the abbreviation QUB has been defined (page 6). This should be rectified.	Thank you – we have described this in full.
A brief description of data analysis after download from MS forms should be included.	Thank you – we have detailed our analysis section from data obtained from the MS forms questionnaire in the data analysis section.
The methodology behind the selection (or exclusion) of definitions presented to the focus groups needs to be described. For example, was selection based on a previous review, or on database searches, or something else?	We have added the following the methods section "Our starting point was to bring existing definitions to the focus group in addition to the development of a new definition based on discussion. Existing definitions were based on a previous systematic review (Leonard et al., 2025). However, this was an iterative process, whereby if the expert participants in the focus group expressed that a current definition was not captured, the team would add that to discussion."

One limitation of the questionnaire is that whilst participants ranked the definitions in order of preference, there does not seem to have been a question as to whether respondents actually endorsed the top ranked definition, only that it was the most preferred of the definitions presented. One could argue that this gives sufficient proxy for reaching consensus, but it's not quite the same as >=80% of respondents giving a clear endorsement.	Our criteria was >80% consensus i.e. 80% or above agreed that this was the most preferred definition of complex case management.<br>We also collected open ended responses on in round 1 on each definition.
Results: 18% of respondents had a professional background of "other". This is missing from the text. What were these "other" professional backgrounds?	This information was not collected so we are unable to clarify what professional background this 18% came from.
Discussion and conclusions: A discussion of the need for other perspectives on the definition of complex case management is not included. This article gives the perspective of professionals working as complex case managers within brain injury, but other stakeholders (patients, families, employers, agencies) have not had the opportunity to give their views or present possible alternative existing definitions. Neither have complex case managers outside membership of this brain injury organisation had the opportunity to participate. A broader dialogue would seem to be needed as a next step, and such points would usefully be included as limitations or next steps.	Thank you for this helpful consideration. We have now added a section at the end of the discussion to suggest that future research consider the further development of the agreed definition through the perspectives of other stakeholders.
It would also be relevant to see more discussion of the specific needs of brain injury survivors in the context of case management, and/or why some patient groups have a need for a case manager, whilst others do not. Cognitive, behavioural and other impairments after brain injury can make it impossible for the individual themselves to drive the necessary contacts and processes with multiple agencies, and information on such aspects would aid understanding.	Thank you for this comment – we think this is an important point and one that we have considered if another paper on the role of case management (see Leonard et al., 2025). For this paper, we believe that it is perhaps beyond the scope to detail why some patients require or don't require a case manager.

VERSION 2 - REVIEW

Reviewer 1

Name Fong, Nicholas

Affiliation UCSF, Anesthesia and Perioperative Medicine

Date 30-Apr-2026

COI

Thank you again for the opportunity to review this manuscript, and for the careful work you have put into the revision. I appreciate the responsiveness to my previous comments, particularly the clarification around the sampling approach across rounds, the correction to the appendix, and the corrected count in the abstract. The added sections to the limitations section also help.

There are a few minor points I would like to raise on the revised version. I want to acknowledge upfront that I may be misreading some of these, and would welcome correction where I am.

1. On the iterative logic of the Delphi process: I think my original concern was perhaps not fully captured in the revision. The added limitations text addresses the fact that Round 1 and Round 2 respondents were not the same individuals, which is helpful. However, if I am reading the methods correctly, Round 2 respondents were also not shown the aggregated group-level feedback from Round 1 before they responded. I believe this is the more substantive departure from a Delphi design, since the iterative logic depends not only on panel continuity but also on participants seeing and reflecting on group results before re-rating. I would find it helpful if the authors addressed this aspect specifically, either by pointing to methodological precedent for it or by adjusting the framing to describe what was achieved (which I still think is meaningful, just not quite what is conventionally meant by Delphi-derived consensus). I also wonder whether the cited sources (Keeney et al. 2006; Avella 2016) actually describe this kind of cross-sectional approach as an accepted modified Delphi variant, or whether they are cited for a related but different point. A brief quote or paraphrase would clarify this.

2. I believe the revision has introduced an internal inconsistency that may confuse readers. In response to my second comment, the authors clarified in the results that no changes were made to the definitions between rounds ("Following review of the qualitative comments, no further changes were made to any definitions"). This is a helpful clarification. However, as far as I can tell, the abstract still states that "two definitions were refined and retained for round two," and the methods section still describes a refinement process ("open questions were analysed and used to revise the two most highly rated definitions" and "used to make minor changes to the definitions for the subsequent round – if required"). If I am reading this correctly, Round 2 was essentially a confirmation vote on unchanged definitions, which is fine, but I think the abstract and methods should be reconciled with the results so that readers come away with a consistent understanding of what happened.

3. On the BABICM conflict-of-interest acknowledgment: I appreciate the added sentence in the limitations. I think it could be slightly strengthened by also acknowledging the role BABICM played in selecting the focus group experts and as a group author, since these are part of what I had originally flagged. The funding and design involvement are noted, but the expert selection and authorship roles are arguably the elements that most directly shape the study's content.

4. A small point: there appear to be a few places in the methods where future or present tense remains from an earlier draft (for example, "a questionnaire will be distributed" and "These were then re-rated in subsequent rounds until stability in responses is observed"). These are minor but worth a final pass of edits.

I think these points are all addressable, and I would be happy to look at a further revision. Thank you again to the authors for their work on this.

**Reviewer**	**2**
**Name**	**Godbolt, Alison**
**Affiliation**	**Karolinska Institute & Danderyd Hospital, Dept of Clinical Sciences and University Dept of Rehabilitation Medicine**
**Date**	**04-May-2026**
**COI**	

The authors have addressed some of my previous points but not all. The most important points are stated below, but I'd suggest that the authors also review my previous points.

The Delphi process (even in modified form) with its iterative design has not really been followed as the questionnaires for the two rounds were answered by different respondents. The study has some value, but should not be described as a Delphi process. Perhaps terms such as ”inspired by Delphi”, or similar, could be considered. As the questionnaire part of the study was not iterative as per Delphi, I considered whether the focus group discussions could instead meet this Delphi recommendation. However, the anonymity requirement for a Delphi panel, which allows less dominant points of view to come forward, was not met by the focus groups.

A modified Delphi process would include anonymised feedback to participants from round 1 before round 2, where round 2 would have the same participants. I do not see that any anonymised feedback was given, for example an anonymised summary of comments given in round 1 provided to the panel (which here is incompletely defined) before round 2. Instead, a new questionnaire seems to have been sent, with different respondents, where respondents to round 2 do not seem to have received any feedback from round 2 other than differences in the presented definitions.

Given that the authors have not given data on the degree of overlap between the respondents to the two questionnaires I assume this data was not collected.

I do not agree with the authors' statement in the discussion that "Our approach aligns more closely with modified Delphi methods….", for the reasons stated above.

The inconsistent use of tenses in the methods (past, future) should be adjusted for clarity.

The degree to which respondents to the questionnaire can be considered experts remains unclear. The authors now include the BABICM website address, which is helpful. From this I see various categories of membership including "practitioner", for members without professional qualifications. This seems to some extent to contradict the authors' description of the BABICM as a membership organisation for professionals. As the questionnaire was sent to all members, respondents would seem to include this category of membership. Given the lack of professional qualifications for the category of membership, this would not seem appropriate. A rationale for the inclusion of members without professional qualifications should be given, or it should be clarified if they were not invited to respond.

That the professions of 18% of respondents are unknown should be acknowledged as a limitation. A understanding of professional competencies of case managers is especially relevant in this new field. It would be reasonable for the authors to have discussed this and have ideas about which professions (or possibly none) this 18% of respondents had, and in the absence of data this would be relevant to included along with discussion of this limitation. Given the lack of information I wonder if this 18% were perhaps members without professional qualifications, which would have consequences (to some extent) for the credibility of the findings.

VERSION 2 - AUTHOR RESPONSE

Reviewer: 1

Mr. Nicholas Fong, UCSF

Comments to the Author:

Thank you again for the opportunity to review this manuscript, and for the careful work you have put into the revision. I appreciate the responsiveness to my previous comments, particularly the clarification around the sampling approach across rounds, the correction to the appendix, and the corrected count in the abstract. The added sections to the limitations section also help.

There are a few minor points I would like to raise on the revised version. I want to acknowledge upfront that I may be misreading some of these, and would welcome correction where I am.

1. On the iterative logic of the Delphi process: I think my original concern was perhaps not fully captured in the revision. The added limitations text addresses the fact that

Round 1 and Round 2 respondents were not the same individuals, which is helpful. However, if I am reading the methods correctly, Round 2 respondents were also not shown the aggregated group-level feedback from Round 1 before they responded. I believe this is the more substantive departure from a Delphi design, since the iterative logic depends not only on panel continuity but also on participants seeing and reflecting on group results before re-rating. I would find it helpful if the authors addressed this aspect specifically, either by pointing to methodological precedent for it or by adjusting the framing to describe what was achieved (which I still think is meaningful, just not quite what is conventionally meant by Delphi-derived consensus). I also wonder whether the cited sources (Keeney et al. 2006; Avella 2016) actually describe this kind of cross-sectional approach as an accepted modified Delphi variant, or whether they are cited for a related but different point. A brief quote or paraphrase would clarify this.

Thank you for comment and concerns around the use of the term Delphi study in our paper. We have taken this on board and clarified throughout that our study is informed by a modified Delphi approach however did not follow these techniques rigidly. We have clarified that this is – firstly, the difference in round one and two samples, and the lack of feedback given to participants before round two.

2. I believe the revision has introduced an internal inconsistency that may confuse readers. In response to my second comment, the authors clarified in the results that no changes were made to the definitions between rounds ("Following review of the qualitative comments, no further changes were made to any definitions"). This is a helpful clarification. However, as far as I can tell, the abstract still states that "two definitions were refined and retained for round two," and the methods section still describes a refinement process ("open questions were analysed and used to revise the two most highly rated definitions" and "used to make minor changes to the definitions for the subsequent round – if required"). If I am reading this correctly, Round 2 was essentially a confirmation vote on unchanged definitions, which is fine, but I think the abstract and methods should be reconciled with the results so that readers come away with a consistent understanding of what happened.

We have now revised the abstract and method to help clarify this process.

3. On the BABICM conflict-of-interest acknowledgment: I appreciate the added sentence in the limitations. I think it could be slightly strengthened by also acknowledging the role BABICM played in selecting the focus group experts and as a group author, since these are part of what I had originally flagged. The funding and design involvement are noted, but the expert selection and authorship roles are arguably the elements that most directly shape the study's content.

Thank you – we have revised the declaration statement and the limitations section to reflect your comment.

4. A small point: there appear to be a few places in the methods where future or present tense remains from an earlier draft (for example, "a questionnaire will be distributed" and "These were then re-rated in subsequent rounds until stability in responses is observed"). These are minor but worth a final pass of edits.

Thank you – we have reviewed the paper and hopefully addressed issues with tenses.

I think these points are all addressable, and I would be happy to look at a further revision. Thank you again to the authors for their work on this.

Reviewer: 2
Dr. Alison Godbolt, Karolinska Institute & Danderyd Hospital
Comments to the Author:
The authors have addressed some of my previous points but not all. The most important points are stated below, but I'd suggest that the authors also review my previous points.

The Delphi process (even in modified form) with its iterative design has not really been followed as the questionnaires for the two rounds were answered by different respondents. The study has some value, but should not be described as a Delphi process. Perhaps terms such as ”inspired by Delphi”, or similar, could be considered. As the questionnaire part of the study was not iterative as per Delphi, I considered whether the focus group discussions could instead meet this Delphi recommendation. However, the anonymity requirement for a Delphi panel, which allows less dominant points of view to come forward, was not met by the focus groups.

A modified Delphi process would include anonymised feedback to participants from round 1 before round 2, where round 2 would have the same participants. I do not see that any anonymised feedback was given, for example an anonymised summary of comments given in round 1 provided to the panel (which here is incompletely defined) before round 2. Instead, a new questionnaire seems to have been sent, with different respondents, where respondents to round 2 do not seem to have received any feedback from round 2 other than differences in the presented definitions.

Thank you for comment and concerns around the use of the term Delphi study in our paper. We have taken this on board and clarified throughout that our study is informed by a modified Delphi approach however did not follow these techniques rigidly. We have clarified that this is – firstly, the difference in round one and two samples, and the lack of feedback given to participants before round two.

Given that the authors have not given data on the degree of overlap between the respondents to the two questionnaires I assume this data was not collected.

Given the surveys were anonymous, we did not collect this data.

I do not agree with the authors' statement in the discussion that "Our approach aligns more closely with modified Delphi methods....", for the reasons stated above.
see response above – in our revision of language.

The inconsistent use of tenses in the methods (past, future) should be adjusted for clarity.

We have reviewed the paper and hopefully addressed all issues with tense.

The degree to which respondents to the questionnaire can be considered experts remains unclear. The authors now include the BABICM website address, which is helpful. From this I see various categories of membership including "practitioner", for members without professional qualifications. This seems to some extent to contradict the authors' description of the BABICM as a membership organisation for professionals. As the questionnaire was sent to all members, respondents would seem to include this category of membership. Given the lack of professional qualifications for the category of membership, this would not seem appropriate. A rationale for the inclusion of members without professional qualifications should be given, or it should be clarified if they were not invited to respond.

Thank you for this important observation – only case managers with professional qualifications including background degrees (e.g. social work, physio) and an accredited case management qualification. We have clarified this in the participant section.

That the professions of 18% of respondents are unknown should be acknowledged as a limitation. A understanding of professional competencies of case managers is especially relevant in this new field. It would be reasonable for the authors to have discussed this and have ideas about which professions (or possibly none) this 18% of respondents had, and in the absence of data this would be relevant to included along with discussion of this limitation. Given the lack of information I wonder if this 18% were perhaps members without professional qualifications, which would have consequences (to some extent) for the credibility of the findings.

As above, only those with previous professional qualifications were included - memberships status registered practitioner or advanced registered practitioner. The "other" option would simply suggest that their previous degree was not on the list of options.

VERSION 3 - REVIEW

**Reviewer**	**1**
**Name**	**Fong, Nicholas**
**Affiliation**	**UCSF, Anesthesia and Perioperative Medicine**
**Date**	**31-May-2026**
**COI**	

Thank you to the authors for their careful revisions. The manuscript has improved substantially, and I appreciate the responsiveness to my earlier comments. Points 1 and 3 have been adequately addressed.

I have two residual concerns, but I believe these are minor enough that the editor can verify the corrections without requiring further review from me.

Point 2 (Refinement inconsistency): The abstract's Design subsection now correctly states that no refinement was required. However, the abstract's Results subsection still reads "two definitions were refined and retained for round two," and the Data Analysis section of the methods still states that open questions were "used to revise" the top two definitions. Both of these contradict the clarification in the results that no changes were made. These should be brought into alignment.

Point 4 (Tense): The Data Analysis section still contains the mixed-tense construction I flagged previously ("These were then re-rated in subsequent rounds until stability in responses is observed"). I also note a small grammatical error that may have been introduced during revision: "An methodological approach" in the abstract should read "A methodological approach."

I recommend acceptance conditional on these minor corrections, which the editor should be able to confirm directly.

VERSION 3 - AUTHOR RESPONSE

Reviewer 1

Point 2 (Refinement inconsistency): The abstract's Design subsection now correctly states that no refinement was required. However, the abstract's Results subsection still reads "two definitions were refined and retained for round two," and the Data Analysis section of the methods still states that open questions were "used to revise" the top two definitions. Both of these contradict the clarification in the results that no changes were made. These should be brought into alignment.

We have now corrected these mistakes.

Point 4 (Tense): The Data Analysis section still contains the mixed-tense construction I flagged previously ("These were then re-rated in subsequent rounds until stability in responses is observed"). I also note a small grammatical error that may have been introduced during revision: "An methodological approach" in the abstract should read "A methodological approach."

We have now corrected these mistakes.
