# Supplementary material for: Defining complex case management in the UK: a modified Delphi approach
Source: BMJ Open. 2026 Aug 17;16(8):e114962. doi: 10.1136/bmjopen-2025-114962 (PMC13504933; doi:10.1136/bmjopen-2025-114962)
Supplement: Supplementary file 3 [file bmjopen-16-8-s002.docx]

Supplementary data S2

Round 1 questionnaire

Thank you for taking the time to complete this questionnaire. Your input is valuable and will help us gain a better understanding of complex case management.

This questionnaire is divided into two parts:

**Part 1** collects basic demographic information.

**Part 2** will get you to rank definitions of complex case management.

Your responses will remain confidential and anonymous. Your anonymised responses will be taken as consent to use this data in analysis, publications and future research.

The estimated time to complete this questionnaire is **approximately 5–10 minutes**.

If you are ready, please proceed to the first section.

Section 1 - Demographic information:

1. Age: ______________
2. Gender:

Male 🞏

Female 🞏

Other 🞏

3. Professional background: ______________
4. Years of experience: ______________

Section 2 - Below are 5 definitions of complex case management. Please rank the definitions from most to least preferred (with 5 the most preferred and 1 the least). Use the boxes below each definition to include why you have ranked the definitions in that particular order (e.g. what you liked or didn’t like about each definition).

Definition 1:

*“A collaborative process, which assesses, plans, implements, coordinates, monitors, and evaluates the options and services required to meet an individual’s health and wellbeing, education and/or occupational needs, using communication and available resources to promote quality, cost-effective, and safe outcomes”*

Rationale for ranking

____________________________________________________________________________________________________________________________________________________________________

Definition 2:

*“Community-based case management is a multidimensional and collaborative process. It involves a set of interventions for assessment, planning, coordinating and review of the options and services required to meet the client’s health related needs, and support them to reach their goals related to participation in life roles.”*

Rationale for ranking

____________________________________________________________________________________________________________________________________________________________________

Definition 3:

*“Case management is a process, encompassing a culmination of consecutive collaborative phases that assist clients to access available and relevant resources necessary for the client to attain their identified goals. Key phases within the case management process include: client identification (screening), assessment, stratifying risk, planning, implementation (care coordination), monitoring, transitioning and evaluation. Within the case management process the Case Manager navigates each phase of the case management process (as applicable) with careful consideration of the client's individual, diverse and special needs, including aspirations, choices, expectations, motivations, preferences and values, and available resources, services and supports.”*

Rationale for ranking

____________________________________________________________________________________________________________________________________________________________________

Definition 4:

*" Complex case management specialises in collaboratively coordinating and overseeing tailored support for clients and their families. Working across health, social care, legal, and community systems, they apply expert knowledge and an extensive professional network to advocate for the client’s best interests. Their role is to navigate complex challenges, deliver creative, person-centred solutions, to support the client's ongoing rehabilitation, independence, and quality of life.”*

Rationale for ranking

____________________________________________________________________________________________________________________________________________________________________

Definition 5:

*“An active process devoted to the coordination, rehabilitation, care and support of people with complex, clinical needs and their families. It aims to facilitate their independence and improve their quality of life whilst acknowledging safety issues.”*

Rationale for ranking

____________________________________________________________________________________________________________________________________________________________________

Are there any areas of research that you feel would help develop the profession and or your work?

______________________________________________________________________________________________________________________________________________________

Further comments

____________________________________________________________________________________________________________________________________________________________________

*Thank you for taking part in this questionnaire*
